# Potential health benefits of lowering gas production and bifidogenic effect of the blends of polydextrose with inulin in a human gut model

**DOI:** 10.3389/fnut.2022.934621

**Published:** 2022-07-29

**Authors:** Liying Zhu, Fangjie Guo, Zeyu Guo, Xiaoqiang Chen, Xiaoguo Qian, Xianglong Li, Xiaoqiong Li, Jinjun Li, Xin Wang, Weiguo Jia

**Affiliations:** ^1^Institute of Food Science, Zhejiang Academy of Agricultural Sciences, Hangzhou, China; ^2^Henan Tailijie Biotech Co., Ltd., Mengzhou, China; ^3^Fengning Pingan High-Tech Industrial Co., Ltd., Chengde, China; ^4^Suzhou Hailu Biotech Co., Ltd., Taicang, China; ^5^The Center of Gerontology and Geriatrics, National Clinical Research Center of Geriatrics, Sichuan University West China Hospital, Chengdu, China

**Keywords:** polydextrose, *in vitro* study, short chain fatty acids, gas production, 16S rRNA gene sequencing, blends of PDX and inulin, bifidogenic effect

## Abstract

Polydextrose is a nutrient supplement, which is widely applied in the food industry. The use of polydextrose in combination with prebiotics and probiotics has recently increased, whereas the fermentation properties of its blend have not yet been fully revealed. We evaluated the metabolic profile of polydextrose, inulin, and their blends by a batch *in vitro* fermentation of fifteen human fecal inocula. After 24 h of fermentation, polydextrose increased the production of gas, ammonia, and several short chain fatty acids, including propionate and butyrate, when compared to its blends, inulin, and fructo-oligosaccharides. Furthermore, polydextrose had the slowest degradation rate of all the carbohydrates tested, consistent with its partial fermentation in the distal colon. The 16S rRNA gene sequencing analysis of the gut microbiome exhibited significantly increased relative abundance of *Clostridium_XVIII*, *Megamonas*, *Mitsuokella*, and *Erysipelotrichaceae_incertae_sedis* in polydextrose compared to other carbohydrates. On the other hand, the blends of polydextrose and inulin (1:1 or 2:1) showed reduced gas production and similar bifidogenicity to inulin alone. The blends not only had similar alpha-diversity and PCoA to inulin but also had a similar abundance of beneficial bacteria, such as *Faecalibacterium* and *Roseburia*, suggesting potential health benefits. Also their low gas production was likely due to the abundance of *Faecalibacterium* and *Anaerostipes*, which were negatively correlated with gas production. Additionally, our *in vitro* fermentation model shows advantages in the large-scale assessment of fermentation performance.

## Introduction

Polydextrose (PDX) is a highly branched glucose polymer with complex random bonds. It was approved 30 years ago as a direct food additive by FDA for use as a nutrient supplement, texturizer, stabilizer or thickener, formulation acid, and humectant (1). Today, it is widely utilized in the food industry as a sucrose substitute (2), a fat replacer (3), or is recognized as a dietary fiber used in healthy foods (4, 5) in a large number of countries.

Early studies have shown PDX offers a variety of health benefits due to the partial fermentation of PDX in the distal colon, such as increasing fecal bulk, softening stools, and reducing fecal pH (6). Recent trials in animals and humans have revealed additional health benefits, such as inducing satiety (7), relief of constipation, improving postprandial serum glucose, protecting against offspring obesity in mouse (8), absorption of minerals from the colon (9), and reducing the risk of developing cancer (10). A clinical trial demonstrated that a combination of PDX with probiotic species *Bifidobacterium animalis* spp. *lactis* 420 reduced waist circumference and food intake (11).

Given the key role of gut microbiota in human health, the effect of human gut microbiota has become one of the important properties of dietary fiber and prebiotics. The microbiota contributes to homeostatic regulation in different tissues in our body (12). Diet is an important regulator of the gut microbiota. Western-style diet, which is low in microbiota-accessible carbohydrates, may irreversibly reduce microbial diversity and lead to the disappearance of specific bacterial species in the digestive system (13). These alterations may result in dysfunctions, contributing to the increase in the development of chronic inflammatory diseases (14). Also, adjustment of the human diet with dietary fiber is recognized to at least in part prevent these diseases (15). As a dietary fiber, the impact of PDX on human gut microbiota remains inconsistent, even on *Bifidobacteria* spp. The stimulatory effect of *Bifidobacteria* spp. of PDX was not only reported in the continuous *in vitro* fermentation study (16) but also in the human clinical trial (17). However, the level of *Bifidobacterium* and *Ruminococcus* species was reported to decline after PDX supplementation by 16S rRNA gene sequencing (18). The changes in the composition of gut microbiota resulting from PDX intervention vary from study to study. Costabile and colleagues reported a significant increase in the butyrate producer *Ruminococcus intestinalis*, *Clostridium histolyticum* (clusters I and II) group, and *Clostridium leptum* (cluster IV) for PDX in comparison to the placebo by qPCR, which failed to detect by fluorescence *in situ* hybridization (19). A human study of 20 healthy adult men using 454 sequencings exhibited the relative abundance of *Faecalibacterium, Phascolarctobacterium, Dialister, Clostridium, and Akkermansia, which was* increased upon PDX consumption, as well as increased abundance of fecal *Clostridiaceae* and *Veillonellaceae*, and decreased abundance of Eubacteriaceae (18). In contrast to these dramatic changes in the composition of the healthy gut microbiota, the results of two other human intervention trials with PDX were relatively monotonic but different. PDX consumption increased the relative abundance of Bacteroidetes in the fecal microbiota of healthy men (20) while increasing Christensenellaceae in overweight or obese individuals (21). Obviously, these disputed results of the effects on gut microbiota do not meet the requirements for the application of PDX as a dietary fiber.

Polydextrose has also been studied as a member of the prebiotics combination in *in vitro* studies (22, 23), animal experiments (24), and human trials (25), the studies of most of which focused on the gas production and *Bifidobacteria* spp. count. Gas is one of the major end products of fermentation by gut microbiota. Also gas production is also one cause of the major adverse effects after dietary fiber and prebiotics intake, such as bloating and abdominal pain (26). PDX is tolerant of microbial fermentation and is, in turn, believed to release less gas (10). However, the results were controversial. Ghoddusin and colleagues exhibited high gas generation in the mixture of PDX with other prebiotics (22), whereas Vester Boler and colleagues reported lower gas production, butyrate, and total SCFAs production in the mixture of PDX with other carbohydrates (23). Although both of the above studies suggested the low bifidogenic capability of the PDX blends, a double-blind, randomized study demonstrated that infant formula with the blend of PDX with GOS showed a bifidogenic effect closer to breast milk than formula without the blend (25). Recently, the Panel of European Food Safety Authority concluded that there is no need for numerical acceptable daily intake for polydextrose after its re-evaluation as a food additive (27). Therefore, further studies on the impact of PDX and its blends on human gut microbiota are needed to meet their increasing application.

In this study, we assessed the health benefits and adverse effects of PDX, inulin, and their blends and their correlation with the gut microbiota by a modified gut model in which the fermentation system is reduced to accommodate large-scale assays. PDX was blended with inulin in two ratios: 1:1(Mix1) and 2:1 (Mix2). Based on the comparative analysis of fermentation metabolites, PDX exhibited a remarkably unique fermentation performance when compared with its blends. The analysis of 16S rRNA gene sequencing revealed the difference in microbial selection and fermentation associated with the metabolites. Our results demonstrated that the potential health benefits of the blends were due to the similarity in both diversity and composition of fermentation microbiota to inulin. Also, the significantly increased relative abundance of *Faecalibacterium* and *Anaerostipes*, which were negatively associated with the gas production, was responsible for the reduced adverse effect of the blends compared to PDX.

## Materials and methods

### Fecal sample origins

Fifteen healthy volunteers aged 25–60 years were recruited for the study. All volunteers consumed traditional Chinese food and no one claimed to be vegetarian. The donors had received neither antibiotics, nor pro- or prebiotic treatments for at least 3 months prior to sample collection. The study protocol was approved by West China Hospital Ethics Committee and registered with the Clinical Trials 2018 No. (286). Fresh fecal samples weighing greater than 3 g were collected into sterilized containers and shipped to the laboratory within 4 h for further analysis.

### Carbon source

Polydextrose was provided by Henan Tailijie Biotech Co., Ltd., Mengzhou, China). The degree of polymerization (DP) distribution was as following: 8.1% of DP < 5, 19.9% of DP 5-10, 65.4% of DP 10-20, and 6.6% of DP > 20. Its molecular weight is approximately 2,130 Da. Fructo-oligosaccharide (VILOF^®^ -NanoFOS P95) and inulin (VILOF^®^ -NanoIN) were provided by Fengning Ping An High-Tech, Chengde, China, of which the percentages of DP 2 to 8 were 93.2 and 45%, respectively. The blends, Mix1 and Mix2, were mixed by PDX1 and inulin in ratios 1:1 and 2:1, respectively. Resistant Dextrin (RD), Fibersol^®^ -2 with 90% of total fiber, was provided by Shanghai Toong Yeuan Food Tech Co., Ltd., Shanghai, China.

### Fermentation media

The basal medium was based on the composition of YCFA described by Duncan (28) and contained the following: 2.5 g/L yeast extract; 10 g/L peptone; 0.8 g/L L-cysteine hydrochloride; 0.05 g/L hemin; 0.9 g/L NaCl; 0.09 g/L MgSO_4_.7H_2_O; 0.09 g/L CaCl_2_.6H_2_O; 0.45 g/L KH_2_ PO_4_, 0.45 g/L K_2_H PO_4_; a mixture of micro vitamins and 0.1 mg/L Resazurin which is the indicator of anaerobic condition. The sole carbon sources (8.0 g/L) were added to YCFA to make the respective growth media. The anaerobic media were prepared by flushing N_2_ and dispensed into 5 to 10 mL serum bottles and autoclaved.

### Fecal batch culture fermentation

Batch culture fermentation was performed similar to the previous study (29). Briefly, fresh fecal samples (0.8 g) were homogenized with 0.1 M phosphate-buffered saline (pH 7.0) using an automatic fecal homogenizer (Halo Biotechnology Co. Ltd., Suzhou, China) to make a 10% (w/v) slurry. Then 0.5 mL of the fecal slurry was inoculated to 5 mL growth media and subject to anaerobic fermentation at 37°C.

After 24 h of fermentation, the air pressure difference of the serum bottle was measured. Subsequently, the broth was aliquoted and centrifuged. The supernatant and precipitation of the broth were stored at −30°C for further metabolic analysis and DNA extraction for qPCR.

### Bacterial gas production measurement

The gas production capacity was determined by the air pressure difference of the serum bottle using a digital manometer (HT1895, Dongguan Xintai Instrument Co., Ltd., Dongguan, China).

### Short chain fatty acids analysis

The concentrations of SCFAs in each broth and its initial feces were determined by gas chromatography (GC) (Shimadzu, GC-2010 Plus, Japan) equipped with a DB-FFAP column (0.32 mm × 30 m × 0.5 μm) (Agilent Technologies, United States) using H_2_ flame ionization detector. The amounts of acetic, propionic, butyric, isobutyric, valeric, and isovaleric acids in the filtrates of the broth and the 10% fecal slurry were measured by the internal standard method with crotonic acid (trans-2-butenoic acid) as the internal standard (30).

### Ammonia determination

Ammonia in feces and its fermentation were determined by colorimetry using the enzymatic method described by Barsotti (31).

### Degradation of the carbohydrates

Degradation of oligosaccharides, such as PDX and inulin, was detected by the thin layer chromatography (TLC) analysis described in the previous study (29). The densities of the oligosaccharide spots in the TLC images were quantified by Quantity One (Bio-Rad, United States). The consumed amounts of oligosaccharides by bacteria were calculated based on the difference in the densities between the medium and the broth. The degradation rate of oligosaccharide was the percentage of the amount consumed to its original amount in the medium.

### DNA extraction

The bacterial genomic DNA of both the feces and the precipitations of fermentation broths were extracted by TIANamp Stool DNA Kit according to the manufacturer’s instructions (TIANGEN BIOTECH, Beijing, China) and quantified by Nanodrop ND-2000 (NanoDrop Technologies, United States).

### Enumeration of *Bifidobacteria* spp. and *Lactobacilli* spp.

The number of *Bifidobacteria* spp. and *Lactobacilli* spp. in the feces and the broth were determined by qPCR using CFX96™ Real-time PCR Detection System (Bio-Rad, United States). Total Bifidobacteria species were measured using specific primers Bifi601F: 5’-GGGTGGTAATGCCGGATG and Bifi601R: 5’-TAAGCCATGGACTTTCACACC-3’ (32). Total Lactobacilli species were measured using specific primers Lac-F: 5’-CACCGCTACACATGGAG-3’ and Lac-R: 5’-AGCAGTAGGGAATCTTCCA-3’ (33). The qPCR condition was based on the previous study (34). Bacterial quantification was performed using standard curves constructed from the known concentrations of a plasmid DNA containing the respective amplicons for each set of primers. The copy number of *Bifidobacterium* amplicons was 5.81 × 10^7^ at 0.2 ng/μL and 5.99 × 10^9^ at 20 ng/μL for *Lactobacilli*.

### 16S rRNA gene sequencing

The V4-V5 variable region of the 16S rRNA gene was amplified using universal primers 515F (5’-GTGCCAGCMGCCGCGG-3’) and 907-R (5’- CCGTCAATTCMTTTRAGTTT-3’). High-throughput sequencing was performed using the Ion Torrent S5 platform at Zhejiang University, China. Operational Units (OTUs) were clustered with a 97% similarity cutoff using USEARCH UPARSE, and the chimeric sequences were removed in the UPARSE pipeline. The phylogenetic affiliation of each 16S rRNA gene sequence was analyzed by USEARCH SINTAX algorithm against the RDP training set (version 18) 16S rRNA database using a confidence threshold of 0.8.

The alpha diversity indices (Chao1, richness, Simpson and Shannon index) and beta-diversity index (Bray-Curtis, Unifrac, Jacaard, and Unifrac-binary distance) were calculated using USEARCH alpha_div and beta_div, respectively. Principal coordinate analysis (PCoA) based on UniFrac-binary distance matrix was plotted by Vegan 2.4.2 and the p value was obtained by permutational multivariate analysis of variance (PERMANOVA). The raw reads count tables at different taxonomic levels (phylum, order, class, family, and genus) were used for differential abundance analysis with DEseq2. Spearman’s rank correlation coefficient was performed to reveal the correlations with the metabolites and the top 50 genera.

### Statistical analysis

The statistical analysis was performed using GraphPad Prism 7.00 software. Significance (*p* < 0.05) was performed by non-parametric tests, of which Friedman test was used for metabolic parameters and Wilcoxon test for alpha-diversity indices and relative abundance.

## Results

In this study, fecal slurries from 15 volunteers were inoculated for the batch *in vitro* fermentation to assess the health effects of PDX, inulin, and their blends: Mix1 and Mix2. Along with the metabolic profiles of the fermentation broths (gas production, SCFAs, ammonia, and degradation rates of carbohydrates), the contents of *Bifidobacteria* spp. and *Lactobacillus* spp. were also analyzed. For comparison, we also tested the well-studied prebiotics, FOS and inulin as well as a potential prebiotic RD with similar DP to PDX.

### Metabolic profile

**FIGURE 1:**
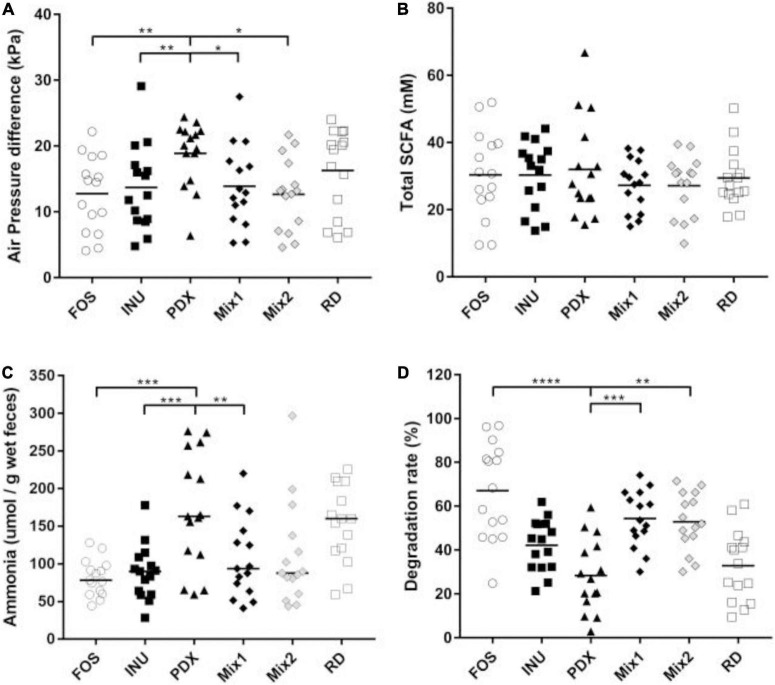
Comparison of metabolic parameters after 24h *in vitro* fermentation. **(A)** The gas productions of different carbohydrate sources were determined by the air pressure difference of the vial. **(B)** The concentrations of the total SCFAs were determined by GC with the internal standard method. **(C)** The concentrations of ammonia in the fermentation broths were determined by colorimetry. **(D)** The degradations of each carbohydrate were determined by TLC. The data of fifteen volunteers are shown in scatter dot plot, the line is at medium. Only the significant differences between inulin (INU) and PDX are shown. *p value < 0.05, **p value < 0.01, ***p value < 0.001, ****p value < 0.0001.

Based on the analysis of the broth after 24 h of anaerobic fermentation, PDX exhibited different metabolic profiles from inulin and FOS, as well as the blends. As shown in Figure 1, PDX produced the most gas, ammonia, and total SCFAs while showing the lowest degradation rate, followed by RD. They are significantly different from the fructans and the blends except for the total SCFAs. PDX produced more gas than FOS, inulin, and the two blends. The ammonia concentration of PDX was higher than the fructans and Mix1 which contained 50% of inulin. The degradation rates of PDX were significantly lower than that of FOS and the two blends. Interestingly, the blends’ degradation rates were higher than both PDX and inulin.

**FIGURE 2:**
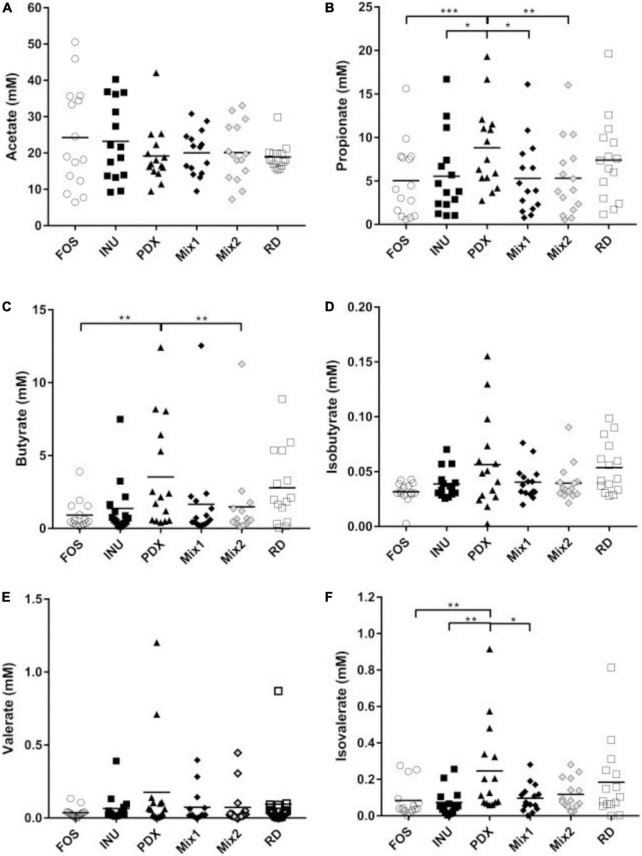
Comparison of individual SCFAs after 24h *in vitro* fermentation. The concentrations of individual SCFAs, including acetate **(A)**, propionate **(B)**, butyrate **(C)**, isobutyrate **(D)**, valerate **(E)**, and isovalerate **(F)** were determined by GC with the internal standard method. The data of fifteen volunteers were shown in a scatter dot plot, line is at mean. Only the significant differences between inulin (INU) and PDX were shown. *p value < 0.05, **p value < 0.01, ***p value < 0.001.

Despite no significant difference in the total SCFAs (Figure 1B), PDX showed the highest production of individual SCFAs among all carbohydrates except for acetate (Figure 2). The amount of PDX in propionate was significantly higher than those of FOS, inulin, and the blends (Figure 2B). As for butyrate, PDX showed significantly stronger production capacity than FOS and Mix2 (Figure 2C). Polydextrose also exhibited significantly higher isovalerate content than FOS, inulin, and Mix1 (Figure 2F). However, the production of acetate in PDX was trending lower compared with FOS, inulin, and the blends but without significance (Figure 2A).

### Effects on the growth of probiotics

**FIGURE 3:**
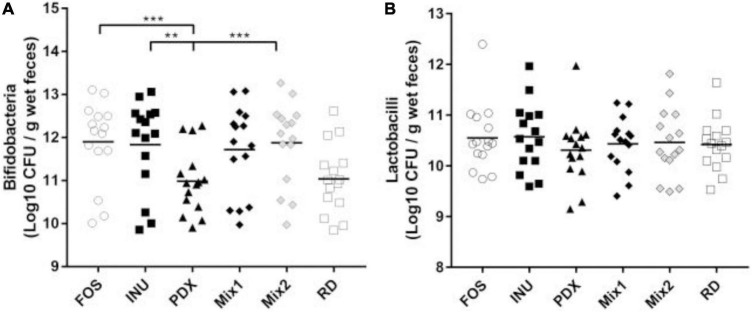
Comparison of *Bifidobacteria spp* and *Lactobacilli spp* after 24h *in vitro* fermentation. The amounts of *Bifidobacteria* spp. **(A)** and *Lactobacilli* spp. **(B)** in the fermented broth of each carbohydrate were determined by qPCR. The data of fifteen volunteers were shown in scatter dot plot, line is at the mean. Only the significant differences between inulin (INU) and PDX were shown. **p value < 0.01, ***p value < 0.001.

To understand the influence of the carbon sources on the growth of *Bifidobacteria spp* and *Lactobacilli spp*, qPCR was performed for the broths after 24 h fermentation. Consistent with the previous report (35), there was no significant difference in lactobacilli growth among carbohydrates (Figure 3B). FOS, inulin, Mix1, and Mix2 stimulated the growth of *Bifidobacteria spp* at a high level, whereas PDX and RD performed at a low level (Figure 3A). The *Bifidobacteria spp* amount in PDX was significantly lower than that in FOS, inulin, and Mix2. These results suggested that the blends of inulin and PDX are bifidogenic.

### Diversity and composition of the microbiota

**FIGURE 4:**
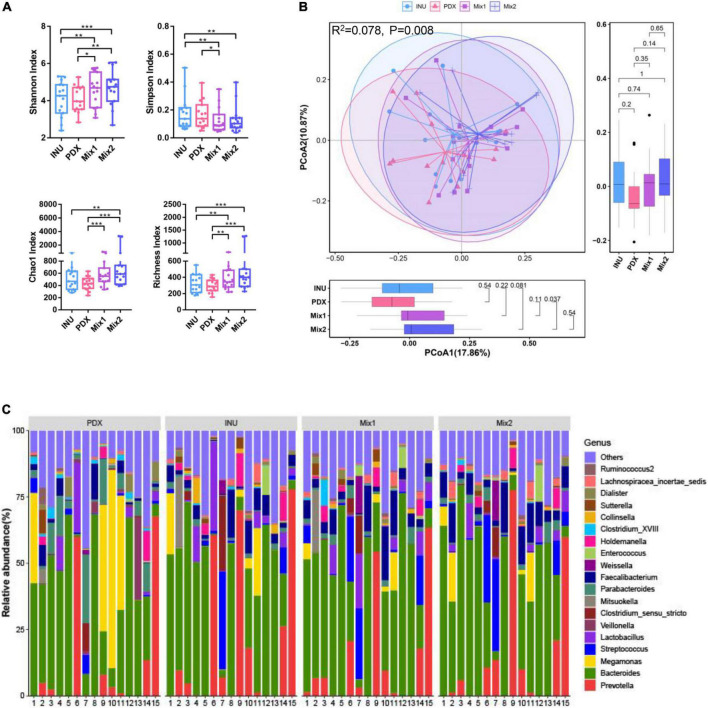
Microbiota comparison of the fermentation broths of inulin, PDX, and their blends. **(A)** The alpha-diversity of the microbiota of the fermentation broths. **(B)** PCoA plot of the fermentation broths with Unifrac-binary distance and the *p* value was calculated by PERMANOVA. **(C)** The microbial composition of the fermentation broths at the genus level. *p value < 0.05, **p value < 0.01, ***p value < 0.001.

To reveal the microbial difference responsible for the significantly different metabolites among PDX, inulin, and their blends, their broths were subjected to 16S rRNA genes sequencing. As shown in Figure 4A, the alpha-diversity indices of the blends of PDX and inulin were higher than both PDX and inulin, but there was no significant difference between PDX and inulin. PcoA plot displayed the overlap of PDX and the blends, which was less than that between inulin and the blends (Figure 4B). Based on the pairwise difference analysis of PCoA1 dimension, the differences between PDX and Mix1 or Mix2 are greater than that between inulin and the blends. This is the same result we see for PCoA2. These results suggest that the microbial community structures of the two blends were close to inulin. As for composition in the genus level, the blends were also similar to inulin, but not PDX (Figure 4C).

### Differences in genus level

**FIGURE 5:**
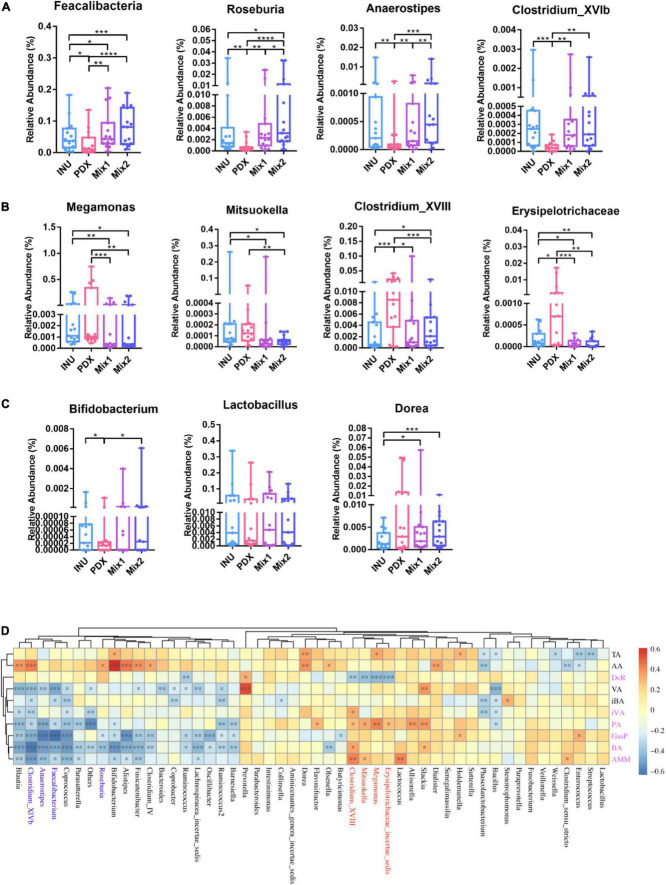
Differential genera between PDX and inulin or their blends and their contribution to metabolites. **(A)** The genera in PDX with 2-fold lower relative abundance than in inulin (INU) or their blends. **(B)** The genera in PDX with 2-fold higher relative abundance than in INU or their blends. **(C)** The genera with a less than 2-fold change abundance between PDX and INU or their blends. **(D)** Heatmap of the correlations with the top 50 genera and the metabolites revealed by Spearman’s rank coefficient correlation analysis. Three genera in panel **(A)** are shown in blue and in panel (B) are in red. The metabolites with significant differences between PDX and INU or their blends were in purple. TA, total SCFAs, AA, acetate, DeR, degradation rate, VA, valerate, iBA, isobutyrate, iVA, isovalerate, PA, propionate, GasP, gas production, BA, butyrate, AMM, ammonia. *p value < 0.05, **p value < 0.01, ***p value < 0.001, ****p value < 0.0001.

The differential abundance analysis revealed that eight genera were significantly different among PDX, inulin, and their blends, with more than 2-fold changes in relative abundance. *Roseburia*, *Anaerostipes*, and *Clostridium_XVlb* (Figure 5A) were significantly decreased in PDX compared to inulin or the blends. *Clostridum_XVIII* and *Erysipelotrichaceae_incertae_sedis* (Figure 5B) significantly increased in PDX compared to the other media. *Mitsuokella* and *Megamonas* (Figure 5B) were significantly lower in the blends than those in PDX or inulin, while there was no significant difference between PDX and inulin. *Dorea* (Figure 5C) was the differential genus between inulin and the blends and the only genus with similar relative abundance between PDX and Mix1 or Mix2. Additionally, *Faecalibacterium* significantly decreased in PDX with the comparison of inulin or the blends (Figure 5A). On the other hand, the relative abundance of *Bifidobacterium* was significantly lower in PDX than in inulin or Mix2. There was no significant difference in *Lactobacillus* abundance among the 4 carbohydrates, despite low amount in PDX. These data confirmed the results obtained by qPCR (Figure 3).

### Correlation with genera and metabolites

Spearman’s rank correlation coefficient analysis was performed to reveal the correlation between genera and metabolites (Figure 5D). The genera enriched in PDX (Figure 5B), *Erysipelotrichaceae_incertae_sedis, Megamonas*, *Mitsuokella*, and *Clostridium_XVIII*, were positively correlated with propionate, ammonia, butyrate, and gas production while negatively associated with degradation rate, which was significant except for degradation rate. In addition, *Clostridium_XVIII* also had a significant positive correlation with isovalerate. The genera reduced in PDX (Figure 5A), *Faecalibacterium*, *Roseburia*, *Anaerostipes*, and *Clostridium_XVlb*, were negatively correlated with gas production, butyrate, propionate, and ammonia. Additionally, *Clostridium_XVlb* showed a significant negative correlation with isovalerate. These results suggest oppositive effects on metabolic parameters between the enriched and reduced genera in PDX, leading to a significant difference in the metabolites between PDX and inulin or their blends (Figures 1, 2).

## Discussion

Polydextrose has been regarded as a food supplement since 1987. Many studies including human trials, animal experiments, and *in vitro* studies, have demonstrated that the ingestion of PDX confers benefits to hosts. In this study, 15 human fecal samples were collected and subjected to batch fermentation with the media containing Mix1, Mix2, PDX, inulin, FOS, and RD as the sole carbohydrate source. PDX exhibited distinct fermentation properties compared to other carbohydrates except for RD. The metabolic and microbial parameters of Mix1 and Mix2, the blends of PDX and inulin, were close to those of inulin but not PDX.

Based on the data from fifteen individuals, PDXs produced a greater increase in propionate (*p* < 0.01) and butyrate (*p* < 0.05) than an equal dose of FOS, inulin, and its blends, which was supported by the recent *in vitro* studies. Romero Marcia and colleagues observed that PDX produced higher levels of butyrate and propionate compared to other soluble resistant glucans in batch fermentation (36). Increased propionate in PDX fermentation was observed in a 3-stage continuous gut model (17) and semicontinuous human colon simulator (37). Propionate and butyrate were reported to regulate energy homeostasis and appetite since they are the most potent agonists for G protein-coupled receptor 41 (GPR41/FFAR3). GPR41 is strongly expressed in the L cell, which is responsible for the release of peptide tyrosine (PYY) and glucagon-like peptide-1 (GLP-1), which is the biomarker or potential biomarker of satiety (38). In addition, GLP-1 has long been recognized as a potent stimulator of insulin secretion (39). Therefore, the significantly high propionate and butyrate observed in this study likely provide an explanation of the health benefits of PDX, such as improving postprandial glycemia and satiety (10, 40).

Besides propionate and butyrate, PDX was found to increase gas production after 24 h fermentation when compared to FOS, inulin, and the blends. This was supported by the results of the 21-day human trial of PDX supplementation, in which subjects taking PDX had the greatest amount of flatulence compared to placebo and soluble maize fiber (23). In fact, the gas production of PDX seems to increase with fermentation time. It has been reported to be low in 12-h fermentation and high after 32 h (22). Lower gas production compared to FOS and inulin in the early time of *in vitro* fermentation was also observed at 4, 8, and 12 h after inoculation (23). The partial fermentation of PDX in the distal colon has been confirmed by both a multiple-stage colon simulator study (41) and a human trial (19), leading to a high mean laxative threshold of approximately 90 g/d (42). Our data indicated that the degradation rate of PDX was observed to be the lowest among all carbohydrates, also suggesting the tolerance of PDX to fermentation. Therefore, the increased gas production after 24 h fermentation indicated the beginning and growing of a delayed fermentation of PDX, which is consistent with the concomitantly detected high amounts of SCFAs. As a result, our *in vitro* study supports that PDX is resistant to microbial utilization in the early stages of fermentation, while produces large amounts of SCFAs, especially butyrate and propionate, in the later stages of fermentation. According to Spearman’s correlation analysis, *Clostridium_XVIII*, *Megamonas*, *Mitsuokella*, and *Erysipelotrichaceae_incertae_sedis* were positively correlated with both propionate and butyrate. *Clostridium* was previously reported to increase after PDX intake compared to the placebo group by both 16S rRNA sequencing (18) and qPCR (19). Clostridial species are producers of propionate (43) and butyrate, in particular, *Clostridium cluster IV* and *XIVa*, such as *Faecalibacterium prausnitzii* and *Eubacterium rectale*, respectively (44). Also *Clostridium_XVIII* was reported to induce homeostatic T-reg responses, as well as *Clostridium cluster IV* and *XIVa* (45), which have been considered the next-generation probiotics (46). Thus, our results implied that *Clostridium_XVIII* was also a producer of propionate and butyrate, at least following the fermentation of PDX. Besides *Clostridium_XVIII*, our data indicated the significantly elevated *Erysipelotrichaceae_incertae_sedis* in PDX compared to both inulin and the blends, which was significantly positively correlated with propionate. Lamichhane and colleagues used the isotopic technique to find that the abundance of Erysipelotrichaceae was positively correlated with the yield of SCFAs derived from PDX, especially of butyrate and propionate (47). The recent *in vitro* study revealed the increased Erysipelotrichaceae in PDX compared to other resistant glucans (36). Erysipelotrichaceae family is reported as SCFAs producers, and some species within this family are capable of producing butyrate (48, 49). It is reported that *Mitsuokella* is a potential genus responsible for the presence of butyric acid in exhaled breath volatile metabolites after chitin-glucan consumption (50). *Megamonas* is regarded as a producer of SCFAs. It produced propionate and acetate in the poultry intestine (51) and positively correlated with fecal lactate in dogs (52) and butyrate in human feces (53). Hence, the significantly increased propionate and butyrate after PDX fermentation in this study is supported by the previous literature. Coinciding with the previous study that several species belonging to Clostridium were ammonia producers (54), *Clostridium_XVIII* exhibited a significantly positive association with ammonia production in this study, leading to the great amount of ammonia after PDX fermentation.

Unfortunately, we did not find the bacteria responsible for gas production, in agreement with the opinion of the previous study that it was difficult to establish the bacteria is responsible for gas production (22). As an alternative, a strongly negative correlation with gas production was found in the predominant genera of inulin compared to PDX, including *Roseburia*, *Anaerostipes*, and *Clostridium_XVlb*. Our data suggested that the inhibition activity could also be responsible for the significant metabolic difference.

In this study, two blends of PDX with inulin were prepared. Although the ratio of PDX in Mix2 (2:1) was higher than that of Mix1 (1:1), both blends produced a lower amount of gas than PDX, which is similar to that of inulin. These results were not in agreement with the prior data that inulin blended with PDX produced the highest amount of gas compared to inulin and PDX alone (22). This might be assigned to the difference in the measurement method and sample size. Besides gas production, other metabolic parameters of the blends were also at the same level as those of inulin but not PDX, such as ammonia, degradation rate, individual SCFAs except for acetate. Furthermore, the analysis of 16S sequencing revealed that not only the microbial community structures but also the compositions of the blends were closer to inulin than to PDX. Among the 8 different genera, only genus *Dorea* showed a significantly different abundance between the blends and inulin. *Dorea* was reported to be positively associated with intestinal permeability (55). Hence, our result suggested the role of inulin in improving colonic barrier function. Mix2 has an increased difference of *Dorea* abundance from inulin than Mix1, suggesting increasing the proportion of inulin in the blend helps reduce intestinal permeability. Both Mix1 and Mix2 increased the relative abundance of *Faecalibacterium*, *Roseburia*, *Anaerostipes*, and *Clostridium_XVlb*, of which the level was comparable to inulin. *Faecalibacterium* is a highly abundant gut microbiota with anti-inflammatory and immunoregulatory properties in healthy human individuals (56). *Roseburia* is an anaerobic bacterium belonging to *Clostridium_XIVa* and plays an important role in maintaining the intestinal barrier function and immune defense (57). Consistent with our result, *Anaerostipes* was reported to increase with *Bifidobacterium* after inulin supplementation (58). Despite the lowest abundance of *Bifidobacterium* in PDX, both Mix1 and Mix2 showed high selectivity toward *Bifidobacterium*, which was comparable to inulin. This was consistent with the previous result that the blends of inulin can augment or maintain their bifidogenic capacity (22). As a result, our results suggested the potential health benefits of the blends of PDX and inulin.

A limitation of this study is the small sample size. However, our results show that the combination of PDX and inulin has the advantage of high bifidogenic capacity and low gas yield. The topic of gas production from the dietary fiber is important because dietary fiber supplementation has emerged as a solution to reduce the health threat posed by Westernization of the diet, while gas production can cause gastrointestinal discomfort and affect the application of dietary fiber. Furthermore, our previous study elucidates increased gas production following inulin fermentation in middle-aged people compared with younger subjects (59). Therefore, further studies are planned in larger cohorts with different age groups, especially the elderly population.

## Conclusion

The fermentation performance of PDX and its blends with inulin were evaluated by the *in vitro* fermentation of the inocula from 15 volunteers. PDX was resistant to being degraded by the gut microbiota to an extent at the beginning of fermentation and enhanced the production of butyrate and propionate later in distal colon, which was likely due to the high abundance of SCFAs producers, *Clostridium_XVIII*, *Megamonas*, *Mitsuokella* and *Erysipelotrichaceae_incertae_sedis*. Also the highest gas production among all carbon sources seemed to result from the significantly reduced abundance of *Faecalibacterium* and *Anaerostipes*, which significantly suppressed the gas production. Despite high gas production and low bifidogenicity of PDX, the blends of PDX with inulin showed reduced gas production and increased bifidogenicity due to their microbiota similar to inulin. Our results suggested the potential health benefits of the blends and the advantages of our fermentation model in characterizing the fermentation properties of prebiotics, especially for large-scale assessment.

## Data availability statement

The datasets presented in this study can be found in online repositories. The names of the repository/repositories and accession number(s) can be found below: https://nmdc.cn/, NMDC40017470.

## Ethics statement

The studies involving human participants were reviewed and approved by West China Hospital Ethics Committee and registered with the Clinical Trials 2018 No. (286). The patients/participants provided their written informed consent to participate in this study.

## Author contributions

ZG did the experiments. FG, XC, and XQ conceived the idea for the study. WJ and XiangL recruited volunteers. XiaoL and JL analyzed the data. LZ and XW designed the experiments. LZ wrote the manuscript. All authors reviewed the manuscript.
